# Sex-Specific Association of Endogenous PCSK9 With Memory Function in Elderly Subjects at High Cardiovascular Risk

**DOI:** 10.3389/fnagi.2021.632655

**Published:** 2021-03-11

**Authors:** Paola G. Simeone, Francesco Vadini, Romina Tripaldi, Rossella Liani, Sonia Ciotti, Augusto Di Castelnuovo, Francesco Cipollone, Francesca Santilli

**Affiliations:** ^1^Department of Medicine and Aging and Center for Advanced Studies and Technology, Chieti, Italy; ^2^Psychoinfectivology Service, Pescara General Hospital, Pescara, Italy; ^3^Mediterranea Cardiocentro, Naples, Italy

**Keywords:** cognitive impairment, PCSK9 (proprotein convertase subtilisin kexin type 9), gender, cardiovascular risk, waist circumference, short term memory

## Abstract

**Background:** Growing evidence indicates that cognitive decline and cardiovascular diseases (CVDs) share common vascular risk factors. Protease proprotein convertase subtilisin/kexin type 9 (PCSK9) is associated with CV disease risk and has been also involved in neuronal differentiation.

**Aim:** Evaluate whether in patients at high CV risk cognitive function is related to PCSK9 levels.

**Methods**. One hundred sixty-six patients (67 female) were enrolled. A detailed neuropsychological (NP) assessment was performed. PCSK9 levels were measured with ELISA.

**Results:** Men had significantly higher short-term memory, executive function, and praxic and mental representation skills, as reflected by Forward Digit Span (FDS) (*p* = 0.005), Trail Making Test-A (TMT-A) (*p* = 0.047), Clock Drawing Test (CDT) (0.016). Endogenous PCSK9 levels were higher in female (*p* = 0.005). On linear regression analysis PCSK9 predicts short term memory only in females (Beta = 0.408, *p* = 0.001), with an interaction between PCSK9 and gender (*p* = 0.004 for interaction PCSK9 by sex). The association of PCSK9 with FDS in female was partially mediated by waist circumference (mediation effect 8.5%).

**Conclusions:** In patients at high CV risk short term memory was directly related to PCSK9 levels only in women, revealing the relevance of sex in this relationship. The association of PCSK9 with memory function may be mediated, at least in part, by waist circumference.

## Introduction

Growing evidence indicates that cognitive decline and cardiovascular diseases (CVDs) share common vascular risk factors, such as smoking, hypertension, high cholesterol, and diabetes mellitus and similar pathogenetic processes such as atherosclerosis and ischemia (Qiu and Fratiglioni, 2015).

Protease proprotein convertase subtilisin/kexin type 9 (PCSK9) is a regulator of low-density lipoprotein (LDL)-cholesterol clearance (Horton et al., 2009), is associated with CVD risk (Seidah et al., 2014) and may have a role in the central nervous system and in neuropsychiatric disorders. PCSK9 has been detected in the brain and in the cerebral spinal fluid (CSF) and has been involved in neuronal differentiation, apoptosis, and inflammation in the brain (O'Connell and Lohoff, 2020).

Few and controversial data are available about the potential role of PCSK9 in Alzheimer Disease (AD): In APOE^(−/−)^ mice fed with a high-fat diet, the hippocampal neuronal apoptosis was associated with an increase of PCSK9 expression (Zhao et al., 2017). Consistently, silencing of PCSK9 attenuated the neuronal apoptosis induced by cerebral ischemia reducing brain damage in mice (Wang et al., 2018). Conversely, PCSK9 may prevent neuronal apoptosis through the decrease in amyloid beta generation (Wu et al., 2014). PCSK9 in the brain is thought to interfere with the cholesterol uptake by neurons, by targeting the VLDL and ApoE receptors, and to impair the amyloid beta clearance, by targeting and degrading the LDL receptor-related protein 1 (LRP1), expressed in microglia, neurons, astrocytes and pericytes, and CD36, mainly present in microglia (Adorni et al., 2019). In contrast, few genetic studies available focused only on PCSK9 genetic variants leading to loss-of-function mutations were not able to show an association between PCSK9 and AD risk (Mefford et al., 2018; Paquette et al., 2018).

Recently, some concerns have been raised about the potential neurological side effects of PCSK9 inhibitors, a class of drugs used as cholesterol-lowering treatments (Mannarino et al., 2018). A meta-analysis found a non-significant trend toward adverse neurocognitive effects for PCSK9 inhibitors in the outcome studies with a larger sample size and longer follow-up (Khan et al., 2017). However, in a randomized trial of the PCSK9 inhibitor evolocumab, no significant between-group difference in cognitive function was observed over a median of 19 months (Giugliano et al., 2017) and 2.2 years of treatment (Gencer et al., 2020), but long-term follow-up studies are required to draw final conclusions.

An additional unexplored issue is the relevance of sex in PCSK9-related cognitive function. Sex-differences have been observed in prevalence and rates of cognitive decline. Namely, women show faster cognitive decline and brain atrophy rates and patterns after diagnosis of mild cognitive impairment (MCI) or AD dementia (Hua et al., 2010; Sundermann et al., 2016). The majority of studies in which cognitive data were stratified by sex in AD dementia indicate that women score lower than men in verbal memory and fluency tasks (Pusswald et al., 2015; Gale et al., 2016).

In a large European cohort (*n* = 3,673), sex has been shown as a strong predictor of PCSK9 and different variables have been associated with PCSK9 in a sex-specific way, e.g., mean corpuscular hemoglobin concentration and smoking habits are PCSK9-independent predictors in women, whereas hypercholesterolemia and physical activity are independent predictors in men (Ferri et al., 2020). Moreover, PCSK9 is regulated by sex hormones and in women PCSK9 correlates inversely with estradiol (E2) (Ooi et al., 2015).

The aim of this study was to evaluate whether, in patients at high CV risk, cognitive function may be related to circulating PCSK9 levels and the possible influence of sex in this association.

## Materials and Methods

### Patients Recruitment

One hundred sixty-six patients (67 female and 99 male) were enrolled in this observational study. For both gender median age was 68 years. Eighty-six patients (32 female and 54 male) had T2DM. T2DM diagnosis was made according to the ADA criteria (fasting plasma glucose ≥ 126 mg/dL or 2-h plasma glucose ≥ 200 mg/dL during OGTT or HbA1c ≥6.5 or a random plasma glucose ≥ 200 mg/dL) (American Diabetes Association, 2020).

Patients were referred to the Clinical research Center of the Center for Advanced Studies and Technology (CAST), “G. D'Annunzio” University Foundation, by primary care physicians or enrolled at the Diabetes Clinic of Chieti University Hospital. Each subject signed written informed consent to participate, and the Protocol was approved by the Ethics Committee of the University of Chieti (Prot.1129 18.07.2013). All the patients were in treatment with low-dose aspirin (100 mg/die) for cardiovascular prevention.

Exclusion criteria were: uncontrolled hypertension, uncontrolled dyslipidemia, significant comorbidities such as kidney or liver disease, pregnancy or lactation, chronic inflammation, cigarette smoking; clinically significant cardiac and/or pulmonary insufficiency; history of malignant neoplasms (diagnosed and treated within the past 5 years); history of malabsorption; regular (daily) alcohol consumption; regular (i.e., more than 3 days per week) non-steroidal anti-inflammatory drug intake.

This study was performed under the Good Clinical Practice regulations (Good Clinical Practice for Trial on Medicinal Product-CPMP/European Commission-July 1990; Decreto Ministeriale 27.4.1992-Ministero della Sanità) and the Declaration of Helsinki (Hong Kong 1989). By signing the protocol, the participants in the study committed to adhere to local legal requirements.

### PCSK9 Levels

All samples were collected at 8 a.m. after an overnight fasting to avoid PCSK9 variations due to its diurnal rhythm (Persson et al., 2010). Blood collected into EDTA-containing vacuum tubes (vacutainer, Becton Dickinson) was centrifuged at 1,200 × g for 10 min at RT to separate plasma. Plasma was aliquoted and frozen at −80°C. PCSK9 levels were measured with commercial enzyme-linked immunosorbent assays (ELISA) kit (#DPC900, R&D) according to the Manufacturer's instructions.

### Neurocognitive Examination

Cognitive function evaluation was evaluated through a short neuropsychological (NP) battery including Forward Digit span (FDS: short-term memory), memory test with 10-s interference (MI: working memory with distracting tasks) Trail Making test part A (TMT-A: executive function and selective attention), Trail Making test part B (TMT-B: executive function and attention switching), Clock Drawing Test (CDT: praxic and mental representation skills). Cognitive tests were taken from the ENB-2 neuropsychological battery, standardized for the Italian population.

The raw scores (raw scores) reported by patients to individual neuropsychological tests were transformed into zeta scores (z-scores) by using averages and standard deviations of the Italian regulatory population.

#### Trail Making Test (Part a and b)

Trail Making Test part A (TMT-A) is a commonly used measure of attention and information processing speed (Lezak, 1995), and already used previously as a measure of attention (Beavers et al., 2017; Tu et al., 2018).

The TMT-B is a well-known instrument for describing the attentive function but at the same time it evaluates sets witching ability working memory and inhibition control (i.e., executive functions); thus, it requires the involvement of executive functions, making it a valid measure of this function (Lezak, 1995; Kemp et al., 2016). Both parts (A and B) of the Trail Making Test consist of 25 circles distributed over a sheet of paper.

#### Forward Digit Span (FDS)

Digit span is the standard test of verbal short-term memory performance that is routinely used in psychological studies, either as a stand-alone test or as part of a number of psychological assessment batteries. Although various other measures of verbal short-term memory capacity exist, the digit span task is the most widely used one in scientific works (Jones and Macken, 2015).

#### Clock Drawing Test (CDT)

The CDT is used for screening for cognitive impairment and dementia and as a measure of spatial dysfunction and neglect. It was originally used to assess visuo-constructive abilities but abnormal clock drawing may occur in other cognitive impairments. Doing the test requires verbal understanding, memory, and spatially coded knowledge in addition to constructive skills (Shulman, 2000).

#### Interference Memory Test (IMT: dual task)

Interference memory test is a test that evaluates working memory by memorizing triplets of letters along with a distracting activity (an activity that prevents subvocal repetition) (Mondini et al., 2011).

## Statistical Analysis

Comparisons of variables between men and women were performed by χ^2^ tests or Mann-Whitney U tests. Spearman rank correlation test was used to assess univariable relationships among continuous variables. Multivariable linear regression analysis was performed: (a) to assess the association of memory function (FDS z-score) with PCSK9, separately in men and women; (b) to test if a different association between FDS z-score and PCSK9 occurs in men and women (this hypothesis was tested by adding the interaction term PCSK9^*^sex in the model), and (c) if the association is mediated by waist circumference. We performed causal mediation analysis to verify whether waist circumference is related to the observed PCSK9-memory relationship. Indeed, a test of mediation examines whether the effect of the independent variable (x) on the dependent variable (y) occurs via a third, intervening variable (z). This analysis was suggested by the observation that waist circumference is associated both with PCSK9 and memory function and is a variable likely to be a mediator of the association between PCSK9 and memory function; the hypothesis of mediation was tested and quantified by adding waist circumference in the multivariable model, and evaluating the percentage change in the beta coefficient of the association of PCSK9 with memory function.

Because of a positive skewed distribution, PCSK9 was natural-log-transformed for the analysis. Only 2-tailed probabilities were used for testing statistical significance, and *P* < 0.05 was considered statistically significant. All calculations were carried out using SPSS (SPSS, Chicago, IL, USA).

## Results

### Baseline Characteristics

One hundred sixty-six patients (67 female and 99 male) were enrolled. Baseline characteristics of patients by sex are listed in Table 1.

**Table 1:** Clinical characteristics of study patients by sex.

**Variables**	**Female (*N* = 67)**	**Male (*N* = 99)**	*p*-value^*^
Age (years)	68 (62–71)	68 (63–73)	0.932
Weight (kg)	71 (64–80)	84 (75–92)	** <0.001**
BMI (kg/m^2^)	28.8 (25.7–32.0)	29.0 (26.0–31.2)	0.728
Waist circumference (cm)	99 (91–108)	104 (96–111)	**0.010**
Hip circumference (cm)	108 (102–114)	104 (99–110)	0.067
WHR	0.91 (0.87–0.95)	0.98 (0.94–1.01)	** <0.001**
Systolic BP (mmHg)	139 (130–150)	145 (134–160)	**0.038**
Diastolic BP (mmHg)	74 (68–80)	79 (70–85)	**0.048**
Fasting plasma glucose (mmol/L)	5.44 (5.06–6.56)	5.78 (5.17–7.11)	0.154
HbA1c (%)	6.10 (5.70–6.70)	6.10 (5.60–7.00)	0.986
HbA1c (mmol/mol)	43 (39–50)	6.10 (38–53)	0.986
Creatinine (mg/dl)	53.4 (53.4–61.0)	68.6 (61.0–76.3)	** <0.001**
eGFR (ml/min)	88.6 (68.9–97.7)	89.0 (77.0–98.0)	0.318
Total bilirubin (μmol)	10.3 (8.5–13.7)	12.0 (8.5–17.1)	**0.006**
AST (U/L)	23 (20–28)	25 (20–31)	0.266
ALT (U/L)	28 (24–36)	31 (25–39)	0.167
hs-C-reactive protein (nmol/L)	0.23 (0.11–0.51)	0.18 (0.08–0.34)	0.114
Uric acid (mg/dl)	5.3 (4.4–6.4)	5.6 (4.8–6.7)	0.146
PCSK9 (ng/ml)	308 (251–394)	267 (231–340)	**0.005**
Leptin (ng/ml)^**^	31.0 (15.4–43.0)	18.0 (11.7–34.1)	**0.038**
**Lipid Profile**			
Total cholesterol (mg/dl)	5.15 (4.3–5.6)	4.5 (3.7–5.0)	** <0.001**
Triglycerides (mg/dl)	1.3 (1.1–1.8)	1.4 (1.0–1.9)	0.707
HDL cholesterol (mg/dl)	1.4 (1.1–1.7)	1.22 (1.0–1.4)	**0.001**
LDL cholesterol (mg/dl)	3.0 (2.3–3.4)	2.5 (1.9–3.0)	**0.001**
**Disease**			
T2DM, *n* (%)	32 (47.8%)	54 (54.5%)	0.431
Hypertension, *n* (%)	58 (86.6%)	81 (81.8%)	0.522
Dyslipidemia, *n* (%)	41 (61.2%)	52 (52.5%)	0.339
CVD, *n* (%)	28 (41.8%)	49 (49.5%)	0.346
MS ATP III, *n* (%)	48 (71.6%)	65 (65.7%)	0.498
Stable CAD, *n* (%)	1 (1.5%)	10 (10.1%)	0.052
Previous MI, or revascularization, *n* (%)	6 (9.0%)	18 (18.2%)	0.119
Previous TIA/stroke, o revascularization, *n* (%)	4 (6.0%)	14 (14.1%)	0.128
PAD, *n* (%)	2 (3.0%)	4 (4.0%)	1.000
Microvascular disease, *n* (%)	1 (1.5%)	7 (7.1%)	0.145
**Medications**			
Metformin, *n* (%)	16 (23.9%)	40 (40.4%)	**0.044**
Glinides, *n* (%)	6 (9.0%)	5 (5.1%)	0.354
Sulfonylureas, *n* (%)	1 (1.5%)	3 (3.0%)	0.648
PPAR-gamma, *n* (%)	6 (9.0%)	5 (5.1%)	0.354
GLP1RA, *n* (%)	2 (3.0%)	0 (0%)	0.161
DPP-IVi, *n* (%)	1 (1.5%)	4 (4.0%)	0.649
Acarbose, *n* (%)	0 (0%)	1 (1.0%)	1.000
Insulin, *n* (%)	1 (1.5%)	2 (2.0%)	1.000
SGLT2i, *n* (%)	1 (1.5%)	1 (1.0%)	1.000
ACE-I, *n* (%)	16 (23.9%)	35 (35.4%)	0.126
ARBs, *n* (%)	22 (32.8%)	31 (31.3%)	0.866
Diuretics, *n* (%)	22 (32.8%)	28 (28.3%)	0.606
B-block, *n* (%)	28 (41.8%)	25 (25.3%)	**0.028**
CCA, *n* (%)	16 (23.9%)	26 (26.3%)	0.856
Statins, *n* (%)	27 (40.3%)	47 (47.5%)	0.427
Fibrates, *n* (%)	1 (1.5%)	0 (0%)	0.404
Ezetimibe, *n* (%)	7 (10.4%)	7 (7.1%)	0.571
Proton pump inhibitors, *n* (%)	25 (37.3%)	39 (39.4%)	0.871
ASA, *n* (%)	67 (100%)	99 (100%)	1.000
**Neuropsychological Tests Battery Z-Score**			
Trail making test A	0.59 [0.04–0.95]	0.76 [0.36–1.07]	**0.047**
Trail making test B	0.37 [−0.20–0.70]	0.44 [0.01–0.90]	0.120
Forward digit span	−0.62 [−1.35–0.39]	0.24 [−0.68–0.55]	**0.005**
Clock drawing test	−0.87 [−2.23–0.08]	−0.27 [−1.32–0.47]	**0.016**
Interference memory test	−0.42 [−1.31–0.39]	−0.10 [−0.91–0.69]	0.184

*BMI, body mass index; WHR, waist-hip ratio; BP, blood pressure; MS, metabolic syndrome; CVD, cardiovascular disease; MI, myocardial infarction; TIA, transient ischemic attack; PAD, peripheral artery disease; ACE-I, ACE-inhibitors; ARBs, angiotensin receptor blockers; B-block, beta-blockers; CCA, calcium channel antagonists; ASA, acetylsalicylic acid; MS, Metabolic Syndrome; ATP III, National Cholesterol Education Program—Adult Treatment Panel III*.

** *86 missing, 34 in female and 52 in male*.

*Data are median (25–75th percentile)*.

* *Determined by Mann-Whitney or X^2^ test, as appropriate. Significant values (p < 0.05) are indicated in bold*.

Median age was 68 years and median BMI was 29. Seventy-seven patients (46%; 28 female) had overt CV disease (chronic coronary syndrome, PAD, previous MI, stroke or TIA or revascularizations). Eighty-six patients (52%; 32 female) had T2DM, according to the initial protocol design. Thus, in our population the prevalence of diabetes was higher than in the general population with comparable age (Cho et al., 2018).

In this population at high CV risk, male and female patients displayed differences in a few clinical variables as well in cognitive function parameters and PCSK9 levels. Men had higher weight (*p* < 0.001), waist circumference (*p* = 0.010), WHR (*p* < 0.001), systolic blood pressure (*p* = 0.038), diastolic blood pressure (*p* = 0.048), and creatinine (*p* < 0.001).

Endogenous PCSK9 levels (*p* = 0.005), total cholesterol (*p* < 0.001), HDL (*p* = 0.001), and LDL (*p* = 0.001), circulating leptin (*p* = 0.038) were higher in female (Table 1). Women were less treated with metformin (*p* = 0.044), in line with the literature reporting a higher risk of adverse drug reactions, and possible drug discontinuation, with metformin in women (de Vries et al., 2020) and were more frequently treated with β-blockers (*p* = 0.028), consistently with a recent cohort study (Walli-Attaei et al., 2020). Male had higher executive function, short-term memory, and praxic and mental representation skills, as reflected by TMT-A z-score (*p* = 0.047), FDS z-score (*p* = 0.005), and CDT z-score (0.016) (Table 1).

**Figure 1:**
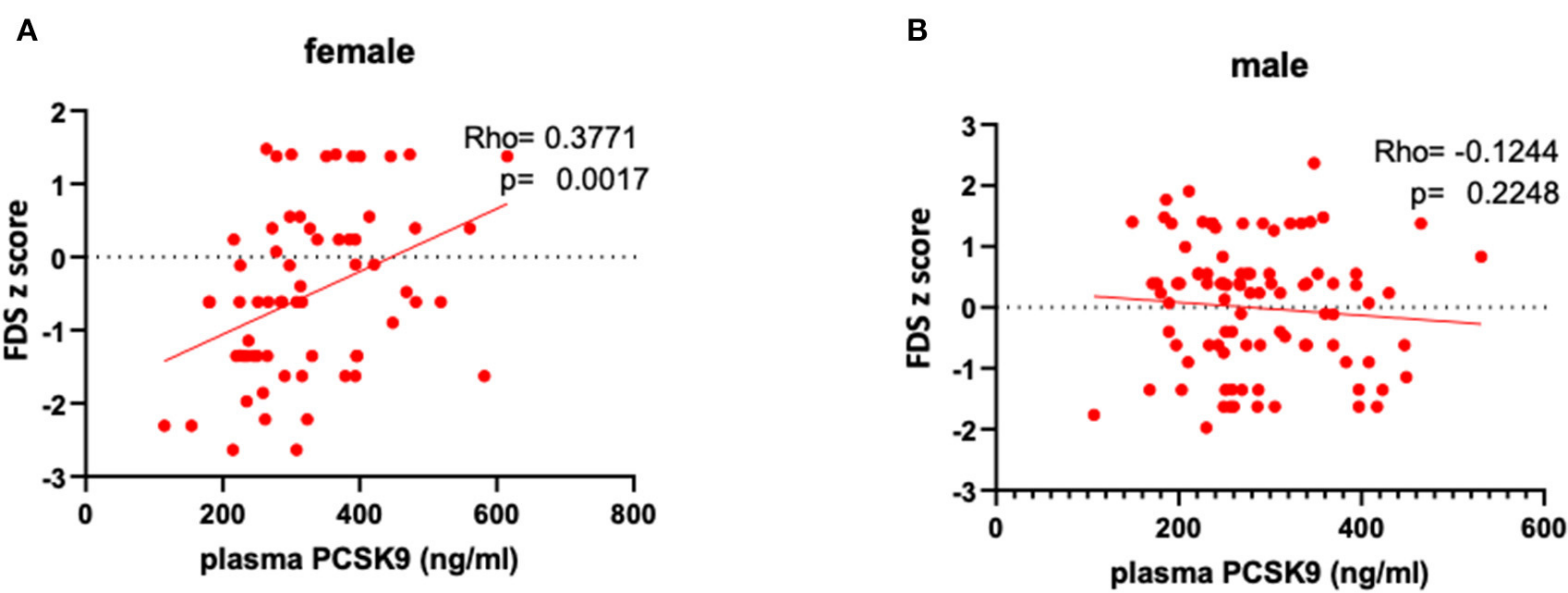
Correlations between circulating PCSK9 levels and short-term memory, as reflected by FDS z-score in female **(A)** and male **(B)** patients.

**Figure 2:**
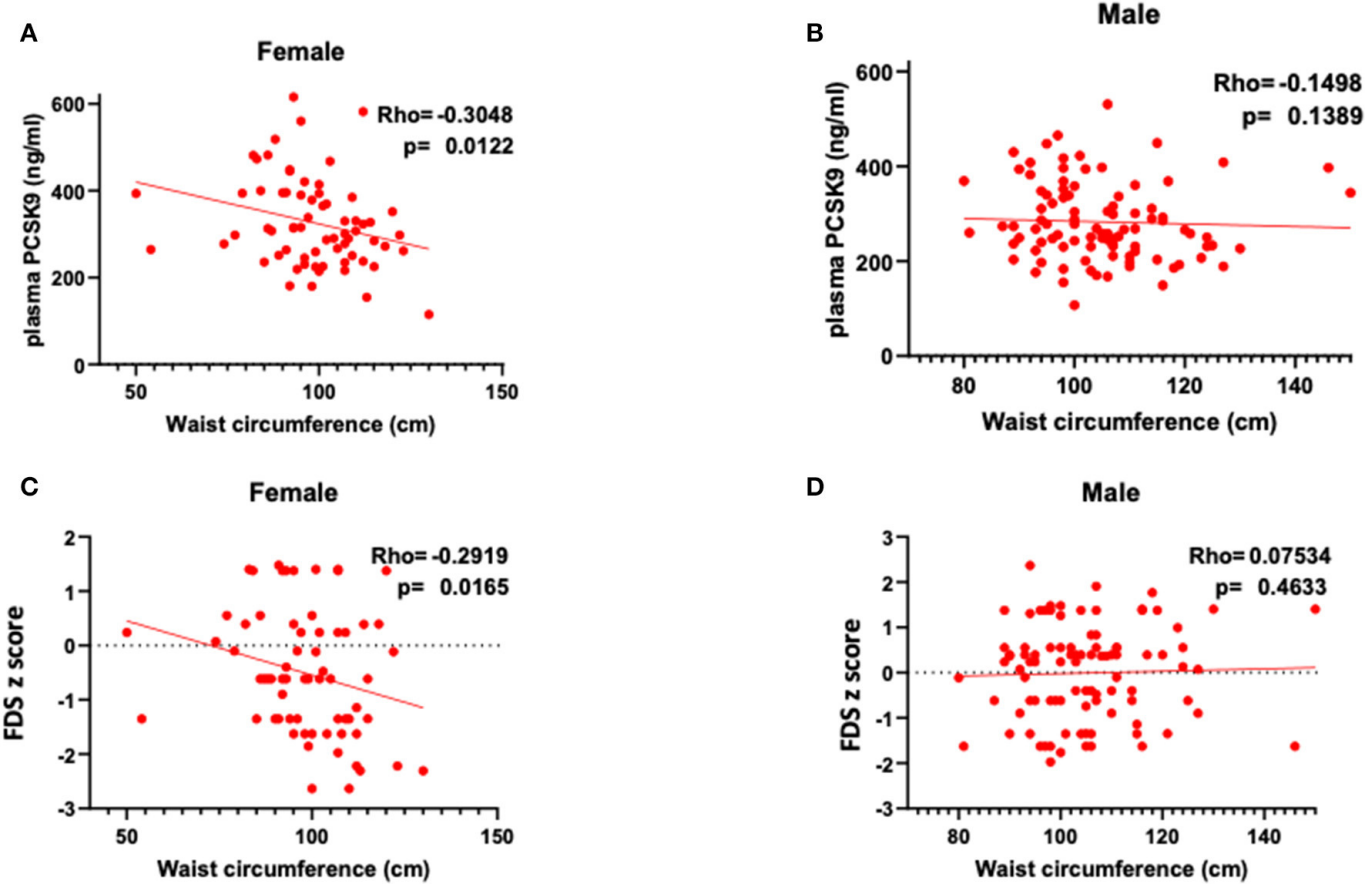
Correlations between waist circumference, circulating PCSK9 levels and short-term memory, as reflected by FDS z-score in female **(A,C)** and in male **(B,D)**.

Circulating PCSK9 levels were directly related to FDS z-score (Rho = 0.377, *p* = 0.002) only in female patients (Table 2 and Figures 1A,B). None of the other tests z-scores were significantly correlated to PCSK9 in either sex. Among the biochemical and anthropometrical variables analyzed, waist circumference was the only variable associated with both FDS z-score and PCSK9 in females (Rho=-0.292, *p* = 0.017 and Rho=-0.305, *p* = 0.012, respectively) (Figures 2A,C). No significant correlation was observed between waist circumference and either FDS z-score or PCSK9 in male (Figures 2B,D).

**Table 2:** Correlations between circulating PCSK9 and cognitive parameters.

			**TMT-A**	**TMT-B**	**FDS**	**CDT**	**IMT**
Female	PCSK9	Rho	0.145	0.051	0.377^*^	0.053	0.118
		*p*-value	0.244	0.727	**0.002**	0.673	0.352
		*N*	66	49	**67**	67	64
Male	PCSK9	Rho	−0.078	−0.058	−0.124	0.094	0.036
		*p*-value	0.448	0.601	0.225	0.364	0.734
		*N*	96	85	97	95	93

*TMT-A, Trail making test A; TMT-B, Trail making test B, FDS, Forward digit span, CDT, Clock drawing test; IMT, Interference Memory Test*.

* *p < 0.05. Significant values (p < 0.05) are indicated in bold*.

Linear regression analysis with FDS z-score as the dependent variable revealed an independent association between LnPCSK9 and FDS z-score only in female (Beta = 0.408, *p* = 0.001) but not in male (Beta=-0.073, *p* = 0.478). The modification of the effect by sex in this association was confirmed by an appropriate test for interaction between PCSK9 and sex, and indeed a significant interaction was found (*p* = 0.002). These findings were independent of a large panel of potential covariates; in fact, they were virtually unchanged following the inclusion in the regression model of total cholesterol or triglycerides, HDL, LDL, systolic and diastolic blood pressure, creatinine, metabolic syndrome, circulating leptin, or ongoing medications affecting PCSK9 levels (Table 3).

**Table 3:** Sex-specific, multivariable association of cognitive performance in the memory domain with PCSK9 in subjects at high cardiovascular risk.

**Variable added to the model**	**Association between LnPCSK9 and FDS z-score in female**		*P* for interaction lnPCSK9*gender
	**Beta**	***P***	
**–**	0.408	0.001	0.002
Triglycerides	0.411	0.000	0.002
Total cholesterol	0.401	0.001	0.002
HDL cholesterol	0.360	0.002	0.003
LDL cholesterol	0.410	0.001	0.001
Leptin^**^	0.523	0.002	0.000
Blood pressure (SAP and DAP)	0.387	0.001	0.003
Creatinine	0.412	0.001	0.002
MS ATPIII	0.405	0.000	0.003
ACE-I	0.427	0.000	0.001
Statins	0.395	0.002	0.002
Ezetimibe	0.397	0.002	0.003

*Data are presented as standardized regression coefficient β. HDL, high density lipoprotein; LDL, low density lipoprotein; SAP, systolic arterial pressure; DAP, diastolic arterial pressure; MS ATPIII, Metabolic Syndrome National Cholesterol Education Program—Adult Treatment Panel III; ACE-I, ACE-inhibitors*.

** *86 missing for leptin concentrations, 34 in female and 52 in male*.

We finally tested the hypothesis that the association between FDS z-score and PCSK9 in female was, at least in part, mediated by waist circumference. We observed that the introduction of waist circumference in the model led to a weakening of the association between LnPCSK9 and FDS z-score in female (Beta from 0.408 to 0.376), thus we quantified that the mediating effect of waist circumference was 8.5%.

## Discussion

In this manuscript we explored the relationship between endogenous PCSK9 levels and cognitive performance in patients at high cardiovascular risk. Since different variables are associated with PCSK9 in a sex-specific way (Picard et al., 2019), and sex-differences have been reported in rates and patterns of cognitive decline, we investigated the influence of sex in this relationship.

Several lines of evidence indicate that PCSK9 may play a role in the pathophysiology of AD both in the pre-symptomatic and symptomatic phases of the disease (O'Connell and Lohoff, 2020; Picard et al., 2019). In autopsy-confirmed human brains, a significant increase in cortical *PCSK9* gene expression and protein levels has been reported in AD patients when compared to age-matched control subjects (Picard et al., 2019). Of note, CSF PCSK9 correlated with CSF tau protein, suggesting that PCSK9 may influence tau metabolism and neurofibrillary tangles accumulation rather than amyloid plaques deposition, at least in the pre-symptomatic phase of late-onset AD (Picard et al., 2019).

A still unanswered question is whether PCSK9 exerts a local effect on the brain or a systemic effect in peripheral tissues thereby affecting the brain, and what is the relationship between circulating and brain PCSK9 concentrations. Neither cholesterol nor PCSK9 cross the blood-brain barrier (BBB) under normal conditions (Dietschy, 2009; Nieweg et al., 2009; Chen et al., 2014); however, several disease states can cause BBB permeability and leakage that might affect brain cholesterol homeostasis. For instance, serum hypercholesterolemia may promote inflammation that damages the BBB and allows passage of LDL, pro-inflammatory cytokines, and other factors into the brain that increase amyloid beta aggregation (Altman and Rutledge, 2010).

PCSK9 is detectable in the CSF of healthy subjects without the typical diurnal pattern of plasma PCSK9, indicating a different regulation in the two body compartments (Chen et al., 2014). Increased CSF concentrations of PCSK9, with a positive correlation with ApoE4 levels, have been reported in AD subjects, suggesting a pathophysiological link between PCSK9, apoE4, and AD (Zimetti et al., 2017).

Thus, circulating PCSK9 can cross the BBB in conditions characterized by inflammation, highly prevalent in our high CV risk population, and modulate cholesterol homeostasis, beta amyloid accumulation, and neuroinflammation. Alternatively, circulating PCSK9 may exert its effects on peripheral tissues in turn contributing to neurocognitive changes. As expected and previously reported (Ooi et al., 2015; Ruscica et al., 2017; Ferri et al., 2020), endogenous PCSK9 levels were higher in female than male. We also found gender differences in patterns of cognitive test performance, as previously observed in other studies (Jorm et al., 2004; De Frias et al., 2006; Munro et al., 2012).

Interestingly, we observed for the first time that PCSK9 and short memory function were directly associated only in female. Consistently, PCSK9 regulation seems to be under tight genetic control, with specific variants of PCSK9 that may pre-dispose to increased AD risk in females only (Picard et al., 2019). In addition, further investigation of these variants in two independent cohorts showed a female specific association with AD risk and with CSF Tau levels in cognitively impaired individuals (Picard et al., 2019).

The pathophysiology underlying the association between PCSK9 and memory function in female is largely uncharacterized. Altered PCSK9 activity in the central nervous system may contribute to the reported deterioration of brain cholesterol homeostasis and indirectly, to lipoprotein dysfunction and AD pathophysiology. Although PCSK9 is a known regulator of LDL cholesterol (Horton et al., 2009) and high LDL and total cholesterol levels have been associated with cognitive impairment in old women (Yaffe et al., 2002), the observation of no correlation between short-term memory and cholesterol levels in our group of patients (data not shown) prevented us to support the hypothesis of an effect mediated by cholesterol lowering.

The influence of sex hormones has been advocated to explain sex differences in both PCSK9 levels (Peticca et al., 2013; Ancelin et al., 2014; Ghosh et al., 2015) and cognitive function, including memory (Mordecai et al., 2008; Joseph et al., 2012). Estrogen levels were inversely correlated to circulating PCSK9 in pre-menopausal females (Ghosh et al., 2015). Maternal serum PCSK9 levels at parturition were significantly elevated in comparison to controls (Peticca et al., 2013). Variation of endogenous estrogen levels during the menstrual cycle likely contributes to the inter-individual variation in PCSK9 and LDL-C in normal females (Ghosh et al., 2015). On the other hand, a longitudinal cohort study revealed gender-specific associations between lipids and cognitive decline in the elderly, involving hormonal status in women (Ancelin et al., 2014). Increased estradiol levels in the late follicular phase in pre-menopausal women was associated with increased activation in left frontal circuitry and decrements in working memory performance (Joseph et al., 2012).

Finally, several lines of evidence including genetic studies indicate that circulating PCSK9 is directly associated with depression, a condition with higher prevalence in women and related to insulin resistance (Nelson et al., 2019; Macchi et al., 2020a).

More intriguing is the hypothesis that the association of PCSK9 with cognitive function in female patients is mediated, at least in part, by its effect on waist circumference. In our cohort, male and female patients, otherwise comparable for most of the clinical characteristics, were significantly different in weight-related anthropometric indices. This was expected since there are large differences in body composition and fat distribution in men vs. women, with women having more body fat and men having a relatively more central distribution of fat (Stevens et al., 2010). This is also reflected by the use of separate waist cut-points by gender (Huxley et al., 2007).

Diverse sources of evidence support a female-specific association between obesity and cognitive impairment. In a longitudinal study obesity has been associated with dementia more strongly in women (Whitmer et al., 2005) and another study showed that, only in women, for every 1.0 increase in BMI at age 70 years, Alzheimer Disease risk increased by 36% (Gustafson et al., 2003). A negative correlation between PCSK9 and central obesity has been previously described in female (Hasan et al., 2017) and both in experimental models and in humans, PCSK9 deficiency results in increased ectopic fat accumulation (Baragetti et al., 2017).

PCSK9 limits visceral adipogenesis likely via adipose VLDLR regulation (Roubtsova et al., 2011).

Vice versa, in conditions of dysfunctional visceral fat depots PCSK9 is induced by leptin and resistin through the involvement of the inflammatory pathway of STAT3. In HepG2 cells, leptin and resistin up-regulated PCSK9 gene and protein expression (Macchi et al., 2020b). However, at least in our cohort, circulating leptin, although higher in women than men, was not related to either PCSK9 or memory function. Similarly, in our population, the prevalence of metabolic syndrome, although previously associated with PCSK9 (Hasan et al., 2017), is comparable between sexes and does not influence the sex-specific relationship between PCSK9 and memory.

PCSK9 induces CD36 degradation thus limiting fatty acid uptake and triglyceride accumulation in tissues such as adipocytes and mouse liver (Demers et al., 2005). PCSK9 regulates the degradation of VLDLR and ApoER2 too, two receptors implicated in both lipid metabolism and neuronal development (Poirier et al., 2008). A role for PCSK9 on sex- and tissue-specific subcellular distribution of VLDLR, has been described (Roubtsova et al., 2015). These data are consistent with our findings showing a mediation effect by waist circumference on the sex-specific association between PCSK9 circulating levels and short-term memory.

Notably, although men and women differed for several cognitive domains, including executive function and praxic and mental representation skills, short term memory was the only cognitive domain significantly associated with PCSK9 circulating levels, consistent with the experimental data indicating a role for PCSK9 in the pathogenesis of AD (Adorni et al., 2019). While sex-differences in most of cognitive functions may be attributed to sex hormones, as discussed above, or to historical differences in education and cognitive reserve (Bloomberg et al., 2021), memory trajectories appear to be independent of education, and diverse mechanisms should be advocated to explain reported differences. Within the limits of our cross-sectional study, we propose that PCSK9 may play a role in this regard.

Limitations of the present study include the small sample size and the cross-sectional nature of our study, which prevented to assess a cause-and effect relationship between PCSK9 and memory function in female. However, the multivariable analysis allowed us to adjust for potential confounders such as the lipid profile and ongoing medications, which are known to affect PCSK9 circulating levels (Macchi et al., 2019). We also have to acknowledge lack of neuro-imaging analysis to detect and characterize cognitive function and the limited neuropsychological battery used to explore cognitive domains. Lack of a genetic analysis for the identification of PCSK9 mutations is an additional limitation, although loss of function and gain of function mutations are relatively rare in Caucasians. Strengths include the study population comprising a well-characterized sample where most of the clinical, anthropometrical, and biochemical features have been addressed in the analysis.

## Conclusions

In conclusion, our results unraveled a previously unappreciated female-specific relationship between PCSK9 circulating levels and short-term memory in patients at high CV risk largely treated with preventive strategies including aspirin and statins. These findings may shed light on the controversial relationship between PCSK9-inhibitors and cognitive function in previous trials. Indeed, the absence of significant between-group difference in changes in cognitive function in randomized trials involving patients who received either PCSK9 inhibitors or placebo may be due to the high prevalence of male patients (approximately 70%) and to lack of sex-disaggregated analyses (Giugliano et al., 2017), while our results reveal a link between PCSK9 and cognition only in female. Thus, sex-specific sub-analyses may be warranted.

## Data Availability Statement

The raw data supporting the conclusions of this article will be made available by the authors, without undue reservation.

## Ethics Statement

The studies involving human participants were reviewed and approved by University of Chieti (Prot.1129 18.07.2013). The patients/participants provided their written informed consent to participate in this study.

## Author Contributions

FS and FV designed and set up the study. PS and FS were involved in patients enrolment. RT, RL, and SC were involved in sample collection and/or analysis. RT and AD performed statistical analysis. FS, FV, RT, PS, and FC were involved in data analysis and interpretation. FS and RT wrote the first draft of the paper. FS, FV, and FC made a critical revision for important intellectual content. FS is the guarantor of this work and, as such, had full access to all the data in the study and takes responsibility for the integrity of the data and the accuracy of the data analysis. All authors contributed to the article and approved the submitted version.

## Conflict of Interest

The authors declare that the research was conducted in the absence of any commercial or financial relationships that could be construed as a potential conflict of interest.

## Abbreviations

CV: cardiovascular
PCSK9: protease proprotein convertase subtilisin/kexin 9
NP: neuropsychological
FDS: Forward Digit Span
TMT-A: Trail Making Test-A
LDL: low-density lipoprotein
CSF: cerebral spinal fluid
MCI: mild cognitive impairment
AD: Alzheimer Disease
T2DM: type 2 diabetes mellitus
ADA: American Diabetes Association
OGTT: Oral glucose tolerance test
ELISA: enzyme-linked immunosorbent assays.
